# Popularizing Wine Tasting Evaluation: An Adaptation of Mouthfeel Terminology

**DOI:** 10.3390/foods15081302

**Published:** 2026-04-09

**Authors:** Lucía Moreno Rodríguez, Andrés Fernández Martín, Ricardo Díaz Armas

**Affiliations:** 1 Department of Business Management and Economic History, University of La Laguna, 38071 San Cristóbal de La Laguna, Spain; lmorenor@ull.edu.es; 2 Department of Economics and Business Management, University of Las Palmas de Gran Canaria, 35017 Las Palmas de Gran Canaria, Spain; andres.fernandezmartin@ulpgc.es

**Keywords:** wine, taste, mouthfeel, sensory evaluation, wine tasting

## Abstract

Wine sensory analysis traditionally relies on complex terminology. This can be challenging to non-expert consumers, particularly regarding mouthfeel sensations. Despite the importance of the latter in determining wine quality and typicity, they lack standardized classification. In this study, we developed and validated a simplified framework for wine taste evaluation that is accessible to consumers with limited tasting experience. The Delphi technique was applied across multiple rounds with a panel of 18 wine experts, primarily sommeliers with experience of diverse consumer profiles. Through an iterative process, attributes were selected from the existing literature and systematically evaluated for relevance, clarity, and accessibility. The validated framework comprises four dimensions: basic tastes (sweetness, acidity, bitterness, salinity, fruitiness); astringency (hardness, dryness, texture); tactile sensations (tingling, warmth, body); and overall evaluation (complexity, balance, taste persistence, alcohol perception). Each attribute includes accessible descriptions and measurement scales anchored with familiar food references to support comparative cognitive processes. All proposed attributes achieved over 85% expert consensus. This framework provides a practical tool that bridges technical wine terminology and everyday consumer language to facilitate communication between industry professionals and consumers. Furthermore, it enables more reliable sensory evaluations in future research and can potentially be extended to other beverages.

## 1. Introduction

Drinking wine is considered by many to be a complex phenomenon due to the numerous physiological and emotional sensations it produces [1,2].

It is known that taste, especially in the case of wine, is the result of multisensory integration [3]. This process involves, on the one hand, sensory stimuli (mainly smell and taste, but also sight, hearing and touch) caused by the volatile compounds responsible for the wine’s aromas and by the tasting environment. On the other hand, it includes the brain processes that are activated in response to these stimuli; that is: emotions, memory, and cognition. All these factors together give rise to flavor, which is therefore unique to each person.

Regarding gustatory stimuli, although a great variety of terms have been defined to describe and evaluate them, there is still no single solid classification in the literature. Gustatory evaluation typically focuses on basic tastes such as sweetness or acidity, while mouthfeel, which is a set of tactile and chemosensory attributes [4,5,6,7,8], receives less attention. Other more global aspects, such as complexity, balance, or taste persistence, are also considered. However, for a wine consumer without training in the field, it can be difficult to understand the terms habitually used to describe these stimuli. Beyond being subjective, they are sometimes redundant and may include hedonic aspects that complicate objective perception [2,5].

In recent decades, several reviews of the terminology used in wine tasting have been carried out [4,5,7,8], specifically regarding mouthfeel sensations. Despite being a set of complex attributes, these sensations are highly relevant for determining perceived quality, typicity, flavor, and enjoyment of wine [8,9]. Since the mouthfeel wheel was devised by Gawel [5], where 53 terms were proposed to describe astringency and other mouthfeel sensations of red wine, different studies have attempted to reduce and simplify the list, but no consensus has been reached. This has resulted in confusion, especially among non-expert consumers.

Therefore, this work aims to adapt the most relevant terms from the sensory–gustatory analysis of red wine to be able to use them in studies involving both experts and novice consumers. Given the diversity and complexity of the attributes resulting from previous studies, this proposal would add value by facilitating gustatory analysis and bringing it closer to all types of consumers.

After reviewing the main works proposing adaptations of the sensory attributes of wine, the Delphi technique was used to define and select the most relevant attributes, as well as to propose the most appropriate way to collect consumer evaluations. This method is recognized for its ability to systematically build consensus among a panel of specialists, making it particularly suitable in contexts where no established criterion exists in the literature [10,11,12]. To this end, we selected a group of wine experts with knowledge and experience in sensory analysis.

## 2. Sensory and Gustatory Analysis of Wine

Taste perception is constructed through the multisensory integration of the stimuli received at the moment of consumption [3].

In the case of wine, in an initial phase, sight provides clues about the quality, style, and origin of the wine [13] based on the clarity, color, or brightness of the liquid, among other factors. Next, stimuli are received from the different aromas in the wine, smell being one of the most widely used senses in wine tasting [1]. Touch is reflected in mouthfeel sensations, both tactile and thermal, but also when holding the glass or bottle. Aspects such as curvature, weight, or the type of glassware play an important role here [14]. Finally, hearing provides extrinsic stimuli from ambient music or noise, if the wine is consumed in company or in a specific setting such as a bar or restaurant, and intrinsic stimuli, caused by the very act of drinking wine, such as the sound of swallowing [15]. All these factors influence the perception of wine tasting, due to the crossmodal correspondences that occur [14,16,17]. Therefore, the gustatory analysis of wine indirectly collects information from the rest of the sensory stimuli perceived at the moment of consumption.

It should be noted, however, that this applies to wine consumption in everyday settings. In controlled sensory analysis, rigorous protocols aim to minimize the influence of other sensory stimuli in order to focus on gustatory and olfactory perception.

According to the classification by Spence & Wang [18], the evaluation of a wine can be hedonic, sensory, analytical, or descriptive. These authors define hedonic evaluation as the consumer’s overall liking (or disliking) of the wine; sensory evaluation as the assessment of the wine’s properties (sweetness, acidity, etc.) and its “impact on the drinker” (astringency, persistence, etc.); analytical assessment as aspects such as the age of the wine (young or aged), complexity, balance, quality, or price; and descriptive assessment as characterizing the wine using adjectives that refer to its character or style (subtle, refined, powerful, etc.), as North [19] concludes in his study.

Spence & Wang [18] distinguish two groups within sensory evaluation: “wine properties” or what are actually known as basic tastes (acidity, bitterness, sweetness, saltiness, etc.) and “impact on the drinker”, which encompasses all the sensations caused by consuming the wine. Other authors have termed this “mouthfeel” [4,8,9]. No consensus has yet been reached regarding the description and classification of wine attributes, which is why our proposal is to redistribute the terms used thus far into three broad categories (basic tastes, mouthfeel, and other global aspects) to simplify sensory–gustatory evaluation by less expert consumers.

### 2.1. Basic Tastes

When it comes to basic tastes, most studies tend to focus on four: sweetness, acidity, bitterness, and saltiness [20,21,22]. Some studies have included umami [16], kokumi [23] and fruity taste [17], although these are less frequent.

Sweetness is one of the first sensations to be noticed, especially on the tip of the tongue, and is mainly due to residual sugar and grape fermentation, although alcohol and glycerol may also contribute. On the other hand, acidity is primarily perceived on the sides of the tongue and inside the cheeks. It is a key attribute for the wine’s balance, providing freshness and preventing sweet wines from becoming cloying [13].

Bitterness usually takes longer to appear compared to other tastes and is perceived with greater intensity on the back and center of the tongue. In red wines, bitterness and astringency are often confused, as both can be caused by the same compounds, such as tannins [24]. However, it should be noted that astringency is defined as the degree of contraction or retraction of the oral surfaces [25], making it a physical reaction to the received stimulus. This corresponds more to a mouthfeel sensation than to a taste [5,8,13,25].

It should be noted, however, that while taste receptors tend to be more abundant in the regions mentioned, they are not exclusively limited to them.

Saltiness is not common in wines, but it is closely associated with perceived salinity, a complex mineral sensation linked to the terroir and the inorganic compounds absorbed during vine development [26,27]. This perception may suggest aspects of salty taste, but it does not correspond to an increased concentration of sodium chloride. Rather, salinity reflects subtle sensory nuances, including minerality and freshness, and is particularly characteristic of wines from coastal regions or vineyards with mineral-rich soils. For example, in wines from the Canary Islands produced in coastal areas, salinity is easier to perceive.

Umami is also not easy to detect and can be confused with the sensation of roundness. It can be perceived in wines containing certain L-amino acids, such as glutamate [21]. Despite its limited relevance in tasting, it can be important in food pairing.

Kokumi has been recently identified as another emerging sensory dimension in wine. It is associated with mouthfulness, continuity and thickness of flavor [23].

While fruitiness is not considered a basic taste, it has been included in some gustatory analyses in previous studies [17]. It is strongly influenced by fruit aromas. From a consumer-oriented perspective, and despite not constituting a basic taste in the strict sense, fruitiness represents one of the most readily identifiable sensory impressions in wine, particularly for non-expert tasters who tend to evaluate flavor as an integrated sensory experience.

However, the gustatory experience extends beyond these tastes. Tactile attributes, referred to as “mouthfeel”, are essential in determining the wine’s style and quality. The overall perception of wine taste emerges from how these basic tastes and mouthfeel sensations interact and balance with one another.

### 2.2. Mouthfeel

Sensory evaluation of wine has progressed beyond analyzing aromas and tastes to encompass a fundamental component: the mouthfeel sensations that consumers experience during tasting.

Mouthfeel is the set of tactile and physical sensations perceived in the oral cavity during wine consumption [6,13,28]. According to Simons [28], chemosensory sensations (such as warmth, burning, coolness, tingling, and numbness) result from the chemical activation of receptors associated with nerve fibers that mediate pain and mechanotransduction. Along these lines, Jackson [13] classified these sensations according to the receptors involved: mechanoreceptors (touch, pressure, vibration), thermoreceptors (heat and cold), nociceptors (pain), and proprioceptors (texture, movement, and position).

Gawel [5] proposed a structured classification of these sensations, known as the “mouthfeel wheel”, which distinguishes two main groups: astringency and tactile sensations. Each of them is divided into sub-qualities, including particulate, surface smoothness, complexity, dryness, dynamic, harshness, green or unripe sensations, weight, warmth, irritation, and texture. These sub-qualities are related to wine compounds such as tannins, acids, alcohol, and their interaction with salivary proteins. Among the most recurrent in the literature are warmth, irritation, astringency, balance, body, texture, and movement [6,8,13,28].

However, this proposal shows some limitations as, despite providing valuable information for describing red wine astringency, some of the terms include a hedonic component in their description and are related to other taste dimensions, for example, “complex”, defined as “a positive hedonic grouping consisting of an amalgam of pleasing astringency sensations, flavor and balanced acidity” [2,5,24].

Likewise, many of these terms are not commonly used in gustatory wine description by consumers. Ortega-Heras [26] shows that most of these terms were not mentioned by any of the 177 judges who participated in their study when they tried to identify which sensory characteristics defined a wine’s typicity, highlighting only acidity, silkiness, and astringency. And although the study by Sáenz-Navajas [29] achieved a reduction to 25 terms, created by experts and general consumers, some of the dimensions from Gawel’s mouthfeel wheel [5] were not reflected, such as irritation (aspects like prickling or tingling) or dynamics (adhesive, grippy, or puckering).

Despite the fact that explicit use of mouthfeel attributes is not widespread, it is possible that some of them are communicated implicitly, without employing the technical terminology seen in the literature. This gap between perception and linguistic expression represents one of the main challenges in wine sensory communication.

For example, Araujo [9] demonstrated that the perceived body of wine comprises sensations associated with three dimensions of mouthfeel: weight, volume, and viscosity. This suggests that non-expert consumers are capable of perceiving and differentiating these sensations, but simply do not know how to express them.

In the case of astringency, non-expert consumers seem to understand its meaning, but the vocabulary they use to describe it is quite limited. Vidal [24] presented 44 terms related to astringency to consumers and asked them to mark those they considered appropriate for describing it. Only 25% of the words were selected by at least 10% of participants. The most relevant terms for referring to astringency were dry, rough, harsh, hard, smooth, and sandpaper.

Furthermore, the inclusion of mouthfeel characteristics in wine sensory analysis is relevant as it contributes significantly to the perceived quality of wine, its structure, and the perception of its taste. In this regard, Araujo [9] shows that mouthfeel aspects such as smoothness, roundness, viscosity, or taste persistence are attributed to higher-quality wine, as opposed to other more negatively perceived aspects, such as harshness. The variety and complexity of these sensations play a fundamental role in the enjoyment and acceptance of a food or beverage and constitute a vital component of the overall consumption experience [4].

Some authors seek to create a universal lexicon to express mouthfeel, but cultural factors make this task difficult [4], not only due to the vocabulary used, but also because of the ability to perceive different sensations based on the habitual diet of each culture. Repeated exposure to certain sensations, such as burning or tingling in the case of spicy foods, can create a certain oral desensitization in populations where this type of food is common. Similarly, cultures with a greater wine-making tradition may have developed a more refined sensitivity toward specific nuances of astringency or texture that remain imperceptible to populations with less exposure to wine.

While there are discrepancies among authors and cultures regarding the sub-qualities that comprise mouthfeel, the main challenge consists in adapting the terminology to language accessible to less expert consumers, regardless of the categories to be defined. This would enable the design of more comprehensible and applicable scales in future studies and allow for larger, more heterogeneous samples. Building on this theoretical framework, the following section outlines the research objectives and questions that guided the study design.

## 3. Research Objectives and Questions

The overall objective of this study is to facilitate the sensory analysis of wine by proposing a classification, description and measurement scale designed specifically for use by consumers with less or nontechnical knowledge of wine tasting. Specifically, this work seeks to answer the following research questions:

- Research question 1: Which sensory taste attributes are most relevant and understandable to consumers with less knowledge of wine tasting?
- Research question 2: How could descriptions be adapted to a lexicon that facilitates the recognition and perception of attributes by consumers with less knowledge of wine tasting?
- Research question 3: How should the perception of the proposed attributes be measured during wine sensory evaluation, ensuring comprehension by consumers?

Additionally, considering the peculiarities of local wines in terms of native grape varieties, terroir, or cultivation methods, it is recognized that each wine-producing region could add specific terms that capture these aspects. Consequently, beyond developing a general framework, this work will propose region-specific adaptations for local wines of the Canary Islands [30], demonstrating how the framework can be contextualized to reflect unique territorial characteristics.

## 4. Materials and Methods

To address these issues, this study proposes developing a wine taste analysis scale, validated by enologists and other experts in the sector using the Delphi technique to simplify the assessment and facilitate the appreciation of wine by non-expert consumers.

**Figure 1:**
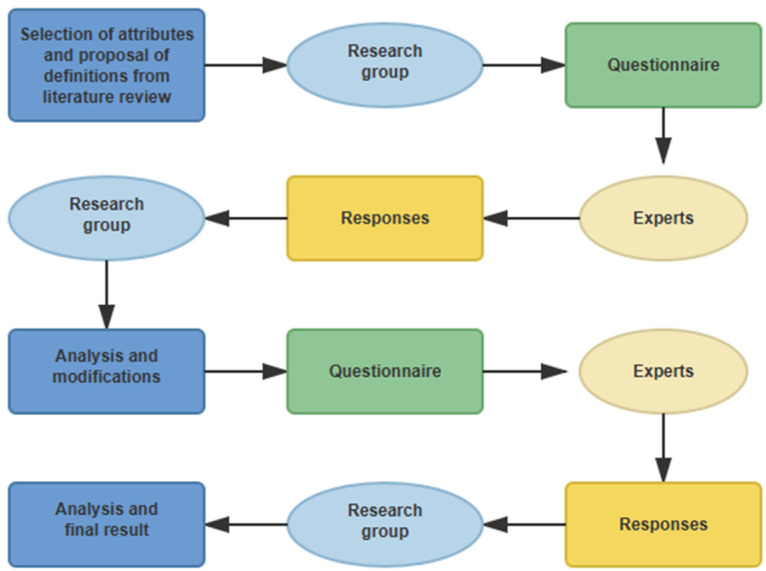
Delphi Technique process. Own elaboration based on [31].

This technique, recognized for its ability to reach consensus among a panel of specialists through an iterative process of successive rounds [11], was applied to rigorously substantiate the adequacy of the technical oenological vocabulary and the measurement scale designed for use in wine tasting with non-expert participants. This process is shown in Figure 1.

We began with an exhaustive review of the literature to identify and compile the most relevant characteristics associated with mouthfeel. On this basis, the initial list was reduced in order to simplify sensory evaluation, and a clear and accessible conceptual description of the term was then proposed.

In a first round, the attributes selected from the literature were presented to the experts through a questionnaire, along with their descriptions and measurement scales. After collecting and analyzing the results of the responses and including some attributes that had not been considered at the outset, a second round was carried out to validate the final results following the modifications made. Although a consensus had been reached, the results were resubmitted after the second round to give participants the opportunity to provide additional input if they wished. However, no further comments or contributions were made in the third round. This iterative process facilitated the exchange of ideas until agreement was reached between the experts and the research team, strengthening the validity and relevance of the results obtained. It is important to note that the implementation of the Delphi technique, which reduces the risk of social pressure in studies of this type, meets four key characteristics: anonymity, iteration with controlled feedback, statistical group response, and expert input [10,12,31].

This methodological approach is expected to culminate in a practical and validated tool for future studies, facilitating the experience and assessment of the general consumer.

### 4.1. Selection of the Expert Panel

To select the panel of experts, we looked for a group of professionals with technical and specialized knowledge of wine. Among the main profiles in the wine sector, such as enologists and viticulturists, sommeliers were identified as the most suitable professional profile for the panel, as they are in direct contact with the consumer. Their work involves advising on and describing wines in language that is understandable and adapted to all types of customers. This ability to transform technical terms and communicate the characteristics of wine makes it possible to evaluate the accessibility and relevance of the proposed terminology.

Thus, an invitation was sent to 100 professionals from the list of active sommeliers in Tenerife. The invitation clearly stated that the objective was to have experts in the sensory analysis of wine who were accustomed to dealing with customers with both high and low levels of knowledge. The invitation was accepted by 27 professionals, but only 18 participants completed the multiple rounds of the process, complying with the considerations in the literature on the established minimum [10,12]. In Table 1, the final composition of the panel can be observed.

In terms of gender, males slightly outnumbered females. Generation X predominated (50%), followed by millennials (27.8%) and baby boomers (22.2%), which is relevant in that it allows for the perspectives of different generations to be considered. In terms of professional profile, although all participants are sommeliers, some are also enologists, wine producers or manufacturers, enriching the diversity of the panel and enabling them to contribute different perspectives.

Finally, in terms of wine consumption habits, practically the entire panel reported regularly consuming local wine (88.9%) and national wine (94.4%), while 77.8% reported regular consumption of international wines.

On one hand, the deep knowledge of local and national wines ensures that the panel is capable of evaluating the proposed terminology considering the specificities of the wines, in this case, from the Canary Islands. On the other hand, the extensive experience with international wines enriches the panel’s perspective and allows terminologies to be identified that have greater potential for universality or, conversely, which are too anchored in specific local traditions.

It should be noted that the panel was informed that responses would be anonymous, in order to avoid bias and social pressure.

### 4.2. Proposed Terminology and Descriptions

While expert sommeliers may be able to discern subtle differences among the seven original categories proposed by Gawel [5], it might be difficult for consumers with less knowledge about wine tasting. By consolidating related sensations into more intuitive categories, we propose a more accessible approach to wine evaluation that also aligns with recent trends in sensory science that emphasize the importance of reducing cognitive load during tasting exercises [32].

Several characteristics were selected from the literature to study their relevance in the taste evaluation of wine. A classification was designed based on four dimensions: basic tastes, astringency, tactile sensations and overall evaluation.

This redistribution responds to criteria such as the frequency with which wine attributes are perceived, prioritizing those most likely to be identifiable by any type of consumer, and semantic clarity, simplifying and clarifying terms that are very similar to each other and could cause confusion.

In terms of *basic tastes*, previous studies identify five basic tastes: sweetness, acidity, bitterness, saltiness and umami [13]. Recent research has also included kokumi, a less established sensory descriptor associated with mouthfulness, continuity and thickness [23]. Sweetness, bitterness, and acidity were selected as the most relevant, given that they are constantly considered in the literature and previous studies that have attempted to reduce the number of attributes [16,21,33].

In Table 2 the selected terms are proposed alongside a brief description detailing the usual location of perception, which serves as a guide to better identify the different tastes.

Salinity, umami and kokumi are considered unusual descriptors in the field of wine [16,23]. Salinity is more prevalent in vineyards in coastal areas, such as islands or Mediterranean regions, due to the minerals absorbed from the soil. Umami, on the other hand, is associated with the amount of glutamate and other chemicals [21]. In the case of kokumi, it has been identified in sparkling wines, but its relevance to other types of wine remains unexplored [23]. Although these attributes can be perceived on certain occasions, it would be very complex for a consumer with less tasting experience, so we decided to discard them based on what has been reported in the literature.

For *mouthfeel*, the study by Gawel [5] is the most extensive in its use of attributes, providing 53 terms that are grouped into astringent and non-astringent sensations. Regarding astringency, they were divided into seven categories: particulate, surface smoothness, complex, drying, dynamic, harsh and unripe.

Thus, in Table 3, we propose a reduction in the assessment of astringency, leaving us with hardness, dryness, and texture.

Hardness (e.g., aggressive, hard) is understood as the grouping of sensations that suggest excessive astringency, such as tightness of the oral surface. Dryness (e.g., dry, numbing), on the other hand, is related to the mucosa of the mouth, such as a sensation of lack of lubrication or saliva. Both sensations are related to the intensity of the tannins. However, maturity (e.g., green, resinous) is associated with high levels of acidity, which, although it can intensify the astringent sensation, is not necessarily implied.

Likewise, although Gawel [5] separates the perception of particulates, their movement in the oral cavity, and the texture of these particulates, these are aspects that are difficult for a non-expert consumer to perceive and differentiate. We propose combining them into a single category—texture, which refers to the sensation of the liquid in the mouth, both in terms of its particulates and its interaction with saliva and movement in the mouth.

Finally, complexity has been defined as a set of perceptions of both tastes and sensations in the mouth and should therefore be considered as an overall assessment not necessarily linked to astringency. A wine could be complex due to the diversity of its elements and enveloping sensation, but not astringent.

On the other hand, regarding non-astringent sensations, considered tactile, studies refer to irritation, warmth or body [5,8], as reflected in Table 4.

Both irritation and warmth are sensations of high perceptual intensity caused by certain components of wine. Alcohol, particularly ethanol, is the main cause of sensations related to heat or burning [13]. Additionally, carbonation in sparkling wines produces tactile stimulation through the bubbles, creating prickling sensations on the tongue and palate. Furthermore, some red wines contain aromatic molecules with spicy notes, such as pepper, which can contribute to these sensations [5,28]. This irritation can range from a subtle tingling to a more pronounced prickling sensation, depending on the concentration of the active compounds and the oral sensitivity of the consumer.

Body, for its part, represents a fundamental dimension in the overall evaluation of wine. In sweet wines, it is often associated with the sugars contained in the wine, while in dry wines it is usually related to alcohol [13].

This term usually comprises three interrelated sub-dimensions: weight (the perceived heaviness or lightness), volume (the sense of fullness or expansion in the mouth), and viscosity (the thickness or fluidity of the liquid). But according to Araujo [9], it is usually communicated in an integrated way in the consumer’s everyday language through expressions such as full-bodied, light or dense wine.

Finally, we grouped together some attributes that can provide an overall assessment of the wine: complexity, balance, taste persistence and alcohol perception (Table 5).

These holistic dimensions differ from the previously described attributes, as they emerge from the integration and interaction of multiple sensory components rather than representing isolated perceptual properties [13]. They require consumers to synthesize information across different sensory modalities and time points, which makes them particularly relevant for understanding consumers’ global evaluations and quality judgments.

This terminology proposal constituted the base material that was subsequently submitted for evaluation using the Delphi method.

### 4.3. Measurement Scales

Furthermore, we consider it necessary to validate the methods used to measure attributes for application in future research. Specifically, we seek to measure experts’ opinions of using interval scales [11] anchored with sensory descriptors that provide meaningful reference points for consumers.

Previous studies have proposed semantic differential scales, ranging from weak to strong/intense for most attributes [2,9]. However, this approach has its limitations. First, the perception of intensity is inherently comparative rather than absolute, since we evaluate each attribute of a wine not in isolation, but in relation to other taste experiences stored in our sensory memory [3]. Second, the terms “weak” and “strong” may be interpreted differently across individuals. Thus, incorporating references to other foods within the questions themselves could help consumers make comparisons and respond more clearly.

To address these limitations, we propose that incorporating references to familiar foods within the scale anchors themselves could help consumers make more accurate comparisons and respond with greater clarity and consistency.

As shown in Table 6, specific food-based examples were proposed for most of the upper extremes (7) of the measurement scales, based on their widespread familiarity, sensory profiles, and relevance to the specific attribute being measured.

These comparisons are not intended to be exact chemical or sensory equivalents, but rather to support the cognitive process consumers experience when forming perceptions of the taste of wine.

### 4.4. Questionnaire Structure

Once we had compiled the terms, definitions and measurement scales to be evaluated, we proceeded to design the questionnaire on the LimeSurvey platform.

For each set of questions, the selected attributes were strategically grouped according to the aforementioned classification (basic tastes, astringency, tactile sensations and overall taste evaluation). This organizational structure was designed to facilitate systematic evaluation and to help experts consider attributes within their sensory category context, potentially improving the coherence and quality of their feedback.

The questionnaire was structured to show each category sequentially, beginning with the most fundamental attributes (basic tastes) and progressing toward more complex and integrative dimensions (overall taste evaluation).

Following usual practice for this type of methodology [10], open-ended questions were included after each block to allow observations and comments. These qualitative inputs proved essential for understanding the reasoning behind acceptance or rejection decisions and for gathering alternative proposals that could strengthen the framework.

The questions were in a multiple-choice format, whereby professionals marked the terms with which they disagreed and then detailed their assessment or proposal. The proposals from each round were analyzed and discussed among the researchers, and when deemed appropriate, incorporated into the revised questionnaire for the subsequent round. This iterative refinement process continued until a high level of consensus was reached. The outcomes of this methodology (consensus rates, panel convergence across rounds, and the resulting validated framework) represent the empirical contribution of the study, fully consistent with its design objectives.

## 5. Results

Given the sample size, attributes that exceeded 85% acceptance by the experts were validated and included. All proposals except irritation met this threshold. Valuable suggestions were gathered regarding the descriptions of some attributes.

Specifically, it was deemed important to note that acidity causes increased salivation, serving as an indicator of its perception. Some studies focus on the interaction between saliva and wine properties [2,34] and this is indeed a clear and noticeable sign for any type of consumer, so the modification was accepted.

Additionally, based on the suggestions, we decided to include fruitiness, which refers to the perception of fruit. It is typically associated with young and fresh wines, though it can also be found in more complex wines depending on grape variety, fermentation techniques, and aging processes. Although it can be considered more as a retronasal aroma than a taste in strict sensory science terms, consumers generally evaluate flavor as a sensory and holistic experience and are often unable to differentiate between aroma and taste when describing their wine experience. Excluding this attribute would mean overlooking a distinguishing factor in wine typicality and, although its application is limited, it adds value to local wines from certain regions where young wine has a stronger social presence, for example, in the Canary Islands. Therefore, we define fruitiness as “the intensity of flavor reminiscent of fruit.”

We also considered adding salinity, which was initially excluded due to its limited relevance in the literature [16,21]. Experts emphasize the importance of the mineral qualities that come from the soil, especially in wines from unique regions with distinctive terroirs, such as the volcanic areas of the Canary Islands or some coastal zones. These geological and geographical particularities can impart distinctive saline characteristics that are intrinsic to the typicality of wines from these areas. To this end, we define salinity as “a salty sensation on the sides of the tongue, linked to the presence of minerals and salts in the wine.” However, we recognize that this is an attribute that would primarily be applicable to wines from these specific regions rather than being universally relevant across all wine types.

The term irritation caused considerable confusion among the experts and was accepted by only 61.1% of participants in the first round. We attribute this result to the fact that it is an attribute encompassing several simultaneous sensations (tingling, tickling, itching) that can be caused by different factors (carbon dioxide or spicy notes such as pepper). This lack of specificity made the term ambiguous and difficult to apply consistently. Additionally, the term carries negative connotations in everyday language, probably derived from its use in the healthcare field where irritation typically implies discomfort, inflammation, or an adverse reaction. The experts’ suggestions proposed replacing it with “tingling,” a descriptor that captures the physical sensation more accurately without implying the aggressive or unpleasant nature associated with irritation.

Finally, several experts noted that complexity not only involves a variety of sensations and tastes, but is also closely related to cultivation methods, production processes, and environmental characteristics that affect wine. In the case of archipelagos or volcanic areas, such as the Canary Islands, volcanic soils rich in minerals and unique microclimates provide distinctive conditions that contribute certain nuances in aromas and tastes, increasing complexity and reinforcing typicality. These factors create layers of flavor that cannot be replicated in other regions. However, when referring only to taste evaluation, it is not possible to directly capture the production process or other factors such as the brand or the shape of the bottle, even though these are known to influence taste perception [29]. Since our framework focuses specifically on the intrinsic sensory properties evaluated through tasting, we maintain the original description of complexity as it relates to the multiplicity of taste sensations perceived, while acknowledging that the full appreciation of wine complexity extends beyond what can be captured through taste alone.

For the measurement scales, although they also met the established acceptance percentage in the first round, some expert opinions were considered and integrated where they enhanced clarity or precision. This included the replacement of several reference terms used as anchors on the scales. For example, using “flat” instead of “simple” at the lower end of the complexity scale. Similarly, “smooth” and “rough” replaced “mild” and “aggressive” when referring to hardness. These changes provide more neutral and texturally descriptive terminology that avoids potentially negative connotations. Regarding taste persistence, experts suggested a simplified measurement approach using “short” and “prolonged” instead of the more verbose “disappears quickly—lingers for a long time.” This modification was accepted as it maintains the same meaning while offering greater conciseness and ease of use during evaluation. Finally, “dark coffee” was added as a more specific descriptor for bitterness, refining the original proposal of simply “coffee.”

Once the modifications had been made and the terms salinity and fruitiness had been included, the revised framework was submitted to the expert panel for the second round of the Delphi process. After this second round, a degree of acceptance of more than 85% was achieved for all attributes, descriptions, and scales, confirming consensus among the experts.

The complete results of this validation process, including the finalized attributes, their definitions, and the associated measurement scales, are reflected in Table 7.

## 6. Discussion

In comparison with previous frameworks that proposed extensive lists of mouthfeel descriptors [5], the present framework prioritises a reduced set of attributes that are more likely to be understood by non-expert consumers.

The development of this sensory evaluation framework using the Delphi technique makes a significant contribution to the field of wine sensory analysis. Unlike some previous studies that have been based on theoretical approaches [5,8,13], this work provides a system of descriptors validated by experts in wine sensory analysis who are accustomed to interacting with all types of consumers, ensuring both scientific rigor and practical applicability.

The rejection of the term “irritation” illustrates the importance of linguistic framing in sensory evaluation. Although the sensation itself is well documented in the literature [5,28], the term may evoke negative associations in everyday language, which could hinder its use in consumer-oriented tasting contexts.

Overall, the proposed framework contributes to bridging the gap between expert sensory terminology and consumer understanding. By prioritising clarity and accessibility, the resulting set of descriptors provides a structured yet simplified approach to wine taste evaluation.

## 7. Conclusions

It should be emphasized that this work constitutes an instrument development phase, providing a validated framework designed for application in future consumer studies.

By focusing on the consumer, this framework simplifies sensory analysis. The inclusion of specific references such as “intense like lemon” for acidity or “thick like syrup” for body reduces the assessment variability and improves the reliability of sensory evaluations.

Furthermore, establishing a common language facilitates communication among enologists, sommeliers, wine critics and consumers, improving transparency in product description while supporting informed consumption and purchasing decisions. This framework can also be applied in sensory marketing strategies.

Additionally, although this study focuses on the taste assessment of wine, it provides a basis for replicating the procedure with other similar products, such as beer, spirits or other beverages, contributing to broader knowledge of sensory analysis.

## 8. Limitations and Future Research

Subjectivity in sensory evaluation remains unavoidable, even when consensus is reached on the main characteristics of each attribute, since flavor is inherently unique to each person due to the multisensory integration process. This is affected by various factors such as saliva composition, emotions, and the physical characteristics of the mouth itself. Quantifying sensations objectively would require advanced instrumental techniques that account for all these factors [1,2,3,34].

Nevertheless, this represents an approach to standardizing terminology that allows these attributes to be applied in future research, especially experimental designs that test practical utility through real wine tastings.

The food-based anchors proposed for the upper extremes of the measurement scales are acknowledged to exhibit natural sensory variability. As these references are intended to activate comparative sensory memory in non-expert consumers rather than to establish precise psychophysical equivalents, this variability does not compromise their cognitive function; however, it should be taken into account when interpreting results in future empirical applications of the framework. Future research could address this limitation by testing alternative or region-specific anchors in applied tasting contexts.

It should be noted that the proposed classification, descriptions, and scales have not yet been empirically validated through real tasting sessions with expert and non-expert consumers. This study aims to provide an accessible framework for wine sensory evaluation and therefore represents a preliminary step before its practical application. Future research will focus on validating the proposed scale in real tasting conditions.

Furthermore, cultural context plays a fundamental role in sensory evaluations; therefore, future research should explore the cross-cultural applicability of these results across other populations.

## Figures and Tables

**Table 1:** Sample characteristics.

		N (18)	(%)
Gender	Female	7	38.9%
	Male	11	61.1%
Generation	Millennials	5	27.8%
	Generation X	9	50%
	Baby boomers	4	22.2%
Professional profile	Winemaker	4	22.2%
	Sommelier	18	100%
	Viticulturist	1	5.6%
	Distributor	1	5.6%
Wine consumption	Local	16	88.9%
	National	17	94.4%
	International	14	77.8%

Own elaboration.

**Table 2:** Basic tastes of wine.

Attribute	Description
Sweetness	Sweet sensation perceived on the tip of the tongue due to residual sugars from fermentation
Bitterness	Bitter sensation perceived at the back of the tongue
Acidity	Refreshing or sharp sensation perceived on the inner walls of the mouth

Own elaboration based on [2,5,25].

**Table 3:** Mouthfeel sensations regarding astringency of wine.

Attribute	Description
Hardness	Excessive and unbalanced astringency, harshness and/or bitterness.
Dryness	Sensation of lack of lubrication in the mouth.
Texture	Sensation of particulates rubbing against the surfaces of the mouth through the movement of the wine and textures perceived when these particulates meet each other.

Own elaboration based on [2,5,25].

**Table 4:** Mouthfeel sensations regarding tactile sensations of wine.

Attribute	Description
Irritation	A sensation of itching, tingling, an effect like bubbles or spices such as pepper.
Warmth	A sensation of warmth and heat produced mainly by the alcohol.
Body	A sensation of weight or density of the wine in the mouth.

Own elaboration based on [2,5,25].

**Table 5:** Overall evaluation of wine tasting.

Attribute	Description
Complexity	Variety and intensity of different tastes and sensations perceived, from an overall flavor experience perspective
Balance	Harmony among all the components of the wine and the sensations they produce, without any standing out above the others
Taste persistence	Duration of the taste and sensations in the mouth after swallowing
Alcohol perception	Prominence of the wine’s alcohol content

Own elaboration based on [2,5,25].

**Table 6:** Measurement scales for wine taste evaluation.

Attribute	Measurement Scale
Sweetness	Low—High, cloying, like honey
Bitterness	Low—High, intense, like coffee
Acidity	Low—High, intense, like lemon
Hardness	Mild—Aggressive
Dryness	Low—High, intense
Texture	Velvety—Grainy
Irritation	Low—High, intense
Warmth	Low—Burning sensation in the throat
Body	Light—Thick, like syrup
Complexity	Simple—Complex
Balance	Some tastes or sensations stand out more—Various nuances equally intense
Taste persistence	Disappears quickly—Lingers for a long time
Alcohol perception	Integrated—Very dominant, burning

Own elaboration based on [2,5,25].

**Table 7:** Classification, description and measurement scales for wine taste evaluation.

Classification		Attribute	Description	Measurement Scale
Basic tastes		Sweetness	Sweet sensation perceived on the tip of the tongue due to residual sugars from fermentation	Low—High, cloying, like honey
		Bitterness	Bitter sensation perceived at the back of the tongue	Low—High, intense, like dark coffee
		Acidity	Refreshing or sharp sensation perceived on the inner walls of the mouth, often accompanied by increased salivation	Low—High, intense, like lemon
		Salinity	Salty sensation on the sides of the tongue, linked to the presence of minerals and salts in the wine.	Low—High, intense, like sea water
		Fruitiness	Intensity of flavors reminiscent of fruit.	Low—High, intense
Mouthfeel	Astringency	Hardness	Excessive and unbalanced astringency, harshness and/or bitterness.	Smooth—Rough
		Dryness	Sensation of lack of lubrication in the mouth.	Low—High, intense
		Texture	Sensation of particulates rubbing against the surfaces of the mouth and meeting each other when the wine is swirled.	Velvety—Grainy
	Tactile sensations	Tingling	Sensation of tingling, tickling or an effect like bubbles or spices such as pepper.	Low—Intense
		Warmth	Sensation of heat produced mainly by the alcohol.	Low—Burning sensation in the throat
		Body	Sensation of weight or density of the wine in the mouth.	Light—Thick, like syrup
Overall taste evaluation		Complexity	Variety and intensity of different flavors and sensations perceived	Flat—Complex
		Balance	Harmony among all the components of the wine and the sensations it produces, without any standing out above the others	Some tastes or sensations stand out more—Various nuances equally intense
		Taste persistence	Duration and intensity of the tastes and sensations in the mouth after swallowing	Short—Prolonged
		Alcohol perception	Prominence of the wine’s alcohol content	Low—Very dominant, fiery

Own elaboration based on [2,5,25].

## Data Availability

The original contributions presented in this study are included in the article. Further inquiries can be directed to the corresponding authors.
